# MRI Dynamically Evaluates the Therapeutic Effect of Recombinant Human MANF on Ischemia/Reperfusion Injury in Rats

**DOI:** 10.3390/ijms17091476

**Published:** 2016-09-05

**Authors:** Xian-Yun Wang, Meng-Meng Song, Si-Xing Bi, Yu-Jun Shen, Yu-Xian Shen, Yong-Qiang Yu

**Affiliations:** 1 The First Affiliated Hospital, Anhui Medical University, 218 Jixi Road, Hefei 230032, China; xyunwang1991@163.com (X.-Y.W.); aysx2015@163.com (S.-X.B.); 2 School of Basic Medical Sciences, Anhui Medical University, 81 Meishan Road, Hefei 230032, China; songmengmeng@ahmu.edu.cn (M.-M.S.); jojo125520@163.com (Y.-J.S.); 3 Biopharmaceutical Institute, Anhui Medical University, 81 Meishan Road, Hefei 230032, China

**Keywords:** MANF, cerebral ischemia/reperfusion injury, MRI

## Abstract

As an endoplasmic reticulum (ER) stress-inducible protein, mesencephalic astrocyte-derived neurotrophic factor (MANF) has been proven to protect dopaminergic neurons and nondopaminergic cells. Our previous studies had shown that MANF protected against ischemia/reperfusion injury. Here, we developed a magnetic resonance imaging (MRI) technology to dynamically evaluate the therapeutic effects of MANF on ischemia/reperfusion injury. We established a rat focal ischemic model by using middle cerebral artery occlusion (MCAO). MRI was performed to investigate the dynamics of lesion formation. MANF protein was injected into the right lateral ventricle at 3 h after reperfusion following MCAO for 90 min, when the obvious lesion firstly appeared according to MRI investigation. T2-weighted imaging for evaluating the therapeutic effects of MANF protein was performed in ischemia/reperfusion injury rats on Days 1, 2, 3, 5, and 7 post-reperfusion combined with histology methods. The results indicated that the administration of MANF protein at the early stage after ischemia/reperfusion injury decreased the mortality, improved the neurological function, reduced the cerebral infarct volume, and alleviated the brain tissue injury. The findings collected from MRI are consistent with the morphological and pathological changes, which suggest that MRI is a useful technology for evaluating the therapeutic effects of drugs.

## 1. Introduction

Stroke is a very common cause of death and disability in the middle-aged and aged populations worldwide [1]. Of all strokes, 87% are ischemic strokes resulting from a disruption in cerebral blood flow in a major brain artery [2]. Tissue plasminogen activator (t-PA) has been proved to be effective for acute ischemic stroke by intravenous administration [3]. Unfortunately, this agent can be used in a very narrow therapeutic window and has some hazardous complications (such as myocardial infarction). Therefore, only few patients can benefit from it [4,5]. Therefore, it is urgently needed to develop new potential treatments for ischemic stroke.

Mesencephalic astrocyte-derived neurotrophic factor (MANF), also named as ARMET, can be induced by endoplasmic reticulum (ER) stress [6]. It has been found that MANF has neuroprotective effects on dopaminergic and nondopaminergic neurons [7,8,9,10]. Pretreatment and treatment after ischemic injury with MANF significantly reduced the ischemia brain injury and improved the behavior in stroke rats [11,12]. In Hoffer’s research, recombinant human MANF was pretreated in middle cerebral artery occlusion (MCAO) rats, and the results indicated that the infarction volume was reduced significantly 2 days after MCAO [13]. In our previous study [14], we intracerebroventricularlly (icv) injected recombinant human MANF protein to the MCAO rats after 2 h of MCAO and found that MANF protein improved the behavior of ischemia rats and promoted the survival of the neuron. However, this injury does not include a reperfusion phase. Nevertheless, the injury caused by reperfusion after cerebral ischemia has been reported that may trigger more severe results [15]. Moreover, since clinical treatment is usually started after the onset of stroke, it is more important to investigate the therapeutic effect of MANF on the injury in reperfusion phase. Another point is that the commonly used neurological function test and 2,3,5-triphenyltetrazolium chloride (TTC) staining cannot be considered as ideal methods for evaluating the therapeutic effects of MANF. Neurological function test usually focuses on behavioral function and cannot reflect the degree of neuron injury. For the latter, the most frequently used tool for measuring the infarction volume is stained by 2,3,5-Triphenyltetrazolium chloride (TTC), which can only detect tissue viability above a certain limit [16]. More unfortunately, TTC staining method usually consumes large amounts of animals because it only represents the infarction at a single time point. In addition, individual differences are also a critical reason why the above two methods are not ideal. Therefore, we have developed a dynamical magnetic resonance imaging (MRI) monitoring technology to evaluate the therapeutic effects of MANF in this study. MRI is a very useful technology in the early diagnosis of cerebral ischemia by a combination of T1- and T2- with diffusion-weighted imaging (DWI) because of its noninvasiveness in both clinical diagnosis and basic research [17,18,19,20]. Additionally, MRI can help demonstrate the dynamic changes occurring in the ischemic lesion and monitor the infarct size in brain, which is valuable to evaluate the time course of the lesion in stroke animals. Many studies have reported to evaluate the change of infarct size in rat cerebral ischemia/reperfusion models using T2-weighted imaging [18,21,22]. As a conventional classical anatomical MR technique, T2-weighted imaging is better in reflecting the change of infarction over a longer period.

In the present study, we carried a dynamic observation on the influence of reperfusion time after cerebral ischemia, which was induced by MCAO for 90 min followed by reperfusion for 1, 2, and 3 h. MANF protein was administrated at a very early time point according to MR scanning with a 3.0 T magnet. The long-term therapeutic effect was also examined dynamically by MRI combined with TTC staining, behavioral tests, and immunohistochemistry method for up to 7 days after intracerebral ventricular injection.

## 2. Results

### 2.1. Effects of MANF Protein on the Mortality, Body Weight, and Neurological Score of Rats with MCAO

**Figure 1:**
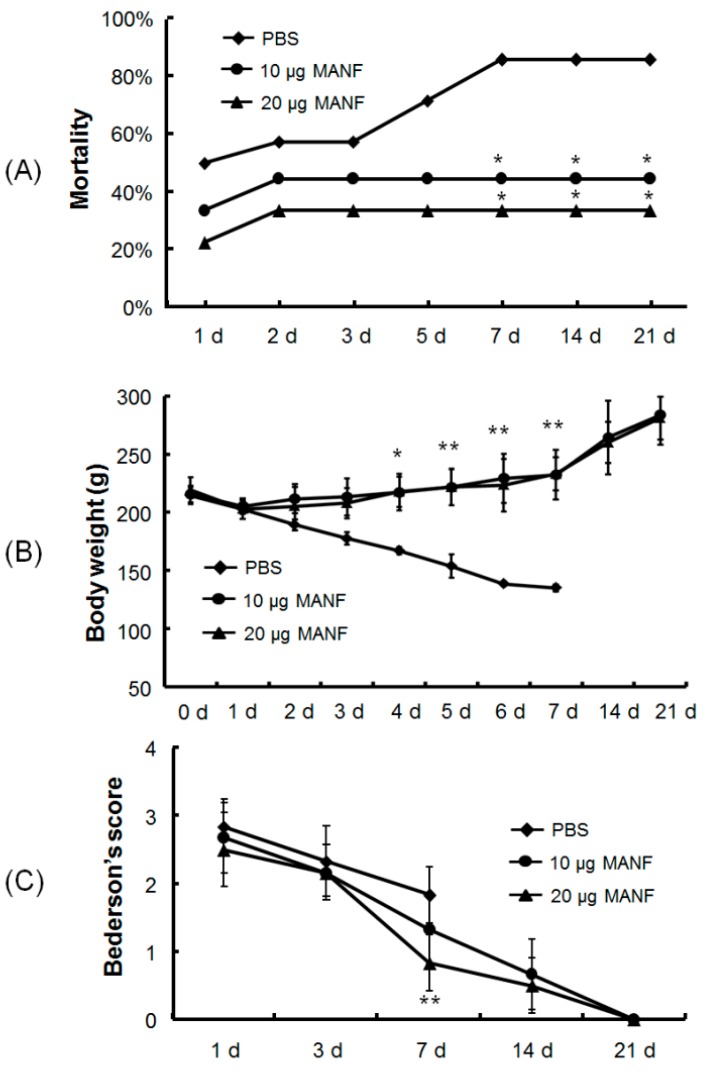
Therapeutic effects of MANF protein on mortalities (**A**), body weight (**B**), and neurological function (**C**) of cerebral ischemia/reperfusion rats. The quantitative data are represented as mean ± SD. * *p* < 0.05, ** *p* < 0.01, compared with PBS group. Note: only one rat in the PBS group survives on the 8th day after MCAO. Therefore, the corresponding body weight in Figure **B** and Bederson’s score in Figures **B** and **C** are not shown. d: day.

In total, 32 rats divided in 3 groups were used for mortality statistics: 18 rats were treated with MANF at 10 μg (*n* = 9) or 20 μg (*n* = 9), and the remaining rats were treated with PBS. In the PBS group, 8 in 14 rats died with mortality of 57.1% within the first 3 days after MCAO due to surgical trauma and severe ischemia. However, in the MANF groups, only 7 in 18 rats died with mortality of 38.8%. Therefore, the rats in the PBS group exhibited a higher mortality compared with that in the MANF-treated group within the first 3 days after MCAO (Figure 1A). Within the 21-day duration of experimentation, rats in the PBS group showed mortality as high as 90%, while rats in the MANF groups had no more death after the first 3 days. The difference in mortality between PBS group and MANF-treated rats is significant from 5th to 21th day post-reperfusion (*p* < 0.05). In the rats treated with 20 μg of MANF, the mortality rate was slightly lower than that with 10 μg of MANF (Figure 1A). These data demonstrated that MANF administration significantly increased the survival of rats with ischemia/reperfusion injury.

The body weight curves of rats in each group at 0, 1, 2, 3, 4, 5, 6, and 7 days after MANF treatment are shown in Figure 1B. All three groups showed an initial decrease on the first day. As time elapsed, the rats administrated with MANF protein exhibited a gentle increase in body weight, while the rats treated only with PBS exhibited a continuous decrease in body weight, and most rats in the PBS group died 5 days after MCAO. During this period, the body weight of rats treated with MANF (10 and 20 µg) increased to a significant value compared with the PBS group (*p* < 0.05).

Neurological function was observed and scored. There was a trend toward better recovery as time extended. After 24 h of reperfusion, there was no difference in the scores reflecting the neurological function in the three groups (2.83 ± 0.41 in the PBS group, 2.67 ± 0.52 in the 10-µg MANF group, and 2.50 ± 0.55 in the 20-µg MANF group). As time elapsed, the scores were significantly reduced in all the groups. Although behavioral deficits in the MANF groups and the PBS group were improved compared with early time points, it was improved much more significantly in the MANF groups. At day 21, the rats in MANF-treated group share a Bederson’s score at 0, which demonstrates that MANF plays a key role in improving neurological function in ischemia/reperfusion rats after administration (Figure 1C).

### 2.2. The Early MRI Manifestation of Ischemia/Reperfusion Injury and the Selection of Proper Time for MANF Administration 

**Figure 2:**
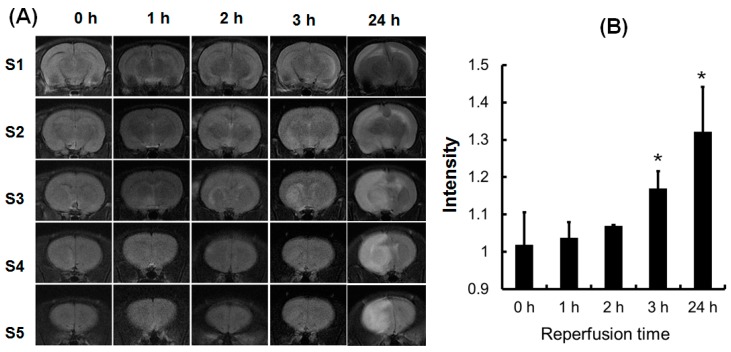
(**A**) Lesion evolusion in T2-weighted images of one representative rat at different time points after reperfusion; (**B**) the quantitative data of Figure **A**. The quantitative values are expressed as mean ± SD of the ratio of ipsilateral to contralateral signal intensity. * *p* < 0.05, compared with the signal intensity at 0 h. h: hour.

At first, we need to determine the proper time for MANF administration by using MRI. After comparison, we found that the lesion was markedly observed at 3 h after reperfusion following 90 min of ischemia as shown in Slice 3 of Figure 2A. The quantitative data in signal intensity are depicted in Figure 2B (the ratio of ipsilateral to contralateral signal intensity). By 1 h after reperfusion, the signal intensity had not significantly changed in the ipsilateral hemisphere. The T2-weighted images obtained at 2 h after reperfusion showed an abnormal signal, but it was not obvious. By 3 h after reperfusion, the high signal areas of T2 abnormalities had expanded and become obvious. However, the lesion was expended almost the entire hemisphere at 24 h post-reperfusion. The signal intensities on T2-weighted images differ significantly along with the time length of ischemia/reperfusion. For 90 min of ischemia, the early signal intensity was significantly increased at 3 h post-reperfusion (*p* < 0.01). Seven rats were used to screen the early MRI manifestation and the time for reperfusion following 90 min ischemia. MRI findings showed that the early lesion was found in 3 in 7 rats at 2 h and at 3 h after reperfusion. These findings suggest that the early edema appeared at 3 h after reperfusion, which provides the basis for the time point of MANF administration.

### 2.3. MRI Manifestation of MANF Restoration on Focal Cerebral Ischemia

**Figure 3:**
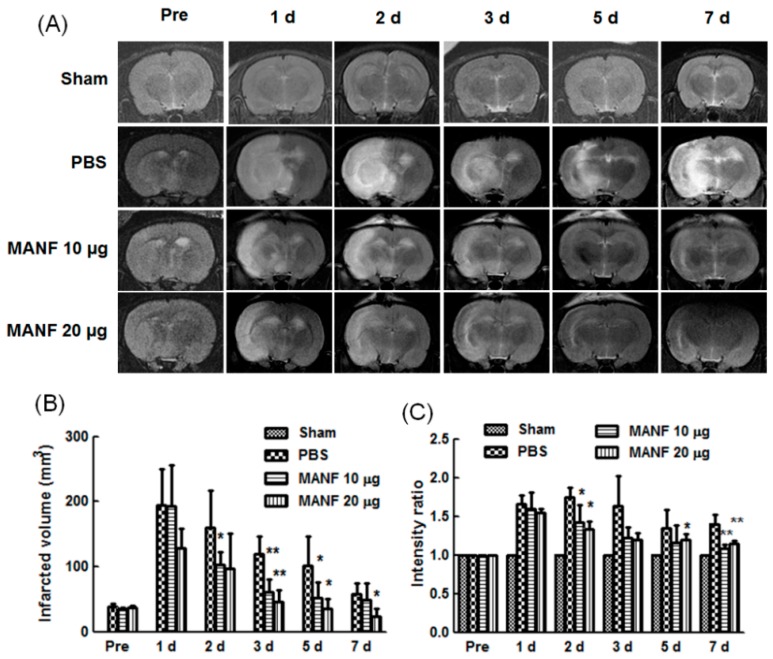
Effect of MANF protein on cerebral infarct volume in rats detected by MRI. (**A**) T2-weighted images in a representative rat in each group before and after MANF protein treatment (1, 2, 3, 5, and 7 days). The quantitative data of infarct volume (**B**) and signal intensities (**C**) on T2-weighted images in each group before and after MANF protein treatment (1, 2, 3, 5, and 7 days). The values are expressed as mean ± SD. * *p* < 0.05, ** *p* < 0.01, compared with PBS group. d: day.

MANF was given at 3 h post-reperfusion following ischemia for 90 min, and the therapeutic effect was monitored by MRI for 7 days. The MRI images showed that MANF (10 or 20 µg) improved the injury induced by ischemia/reperfusion, and 20 µg of MANF was more effective than 10 µg of MANF. The representative images from a rat in each group (PBS, MANF 10 µg, and MANF 20 µg) were shown in Figure 3A, respectively. The infarct zone exhibited a high intensity signal. The intensity volume in the MANF groups was much smaller than that in PBS group after MCAO. Quantitative analysis showed that infarct volume calculated from T2-weighted image in MANF groups was significantly reduced as compared with PBS group (Figure 3B). For the signal intensity, PBS group exhibited an obvious increase compared with MANF group (Figure 3C).

**Figure 4:**
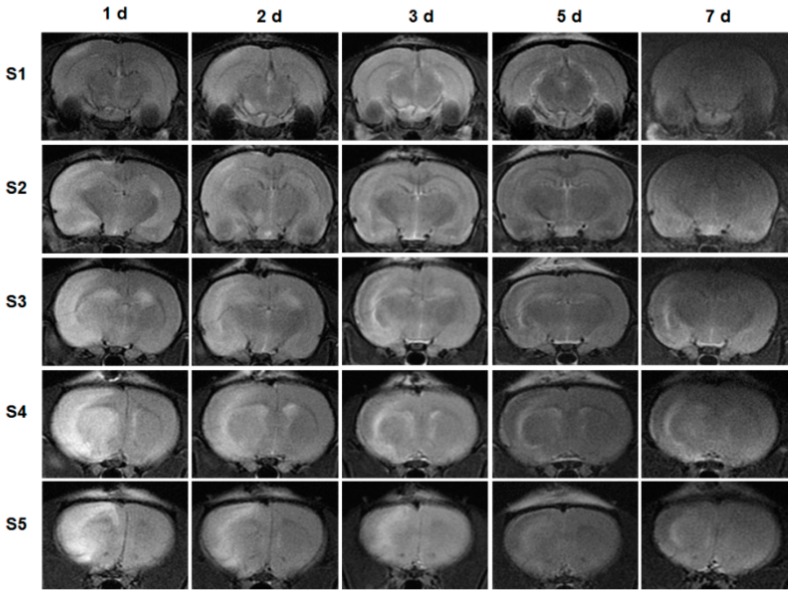
T2-weighted images of 5 brain slices (S1–S5) from a representative rat in the 20-μg MANF group on 1, 2, 3, 5, and 7 days after MANF protein treatment. d: day.

Five slices (S1–S5) on T2-weighted images from a representative rat taken at 1, 2, 3, 5, and 7 days after administration of MANF protein were also shown in Figure 4. On day 1, there was an apparent difference between right hemisphere and left hemisphere, and the isplateral showed obvious high signal intensity. As time prolonged, the high intensity was reduced in 20 μg MANF group, which suggests that the infract area was ameliorated. By day 5, there was no difference between right hemisphere and left hemisphere.

### 2.4. MRI Manifestation Was Confirmed by TTC Staining in Evaluating the Therapeutic Effect of MANF on Focal Cerebral Ischemia

**Figure 5:**
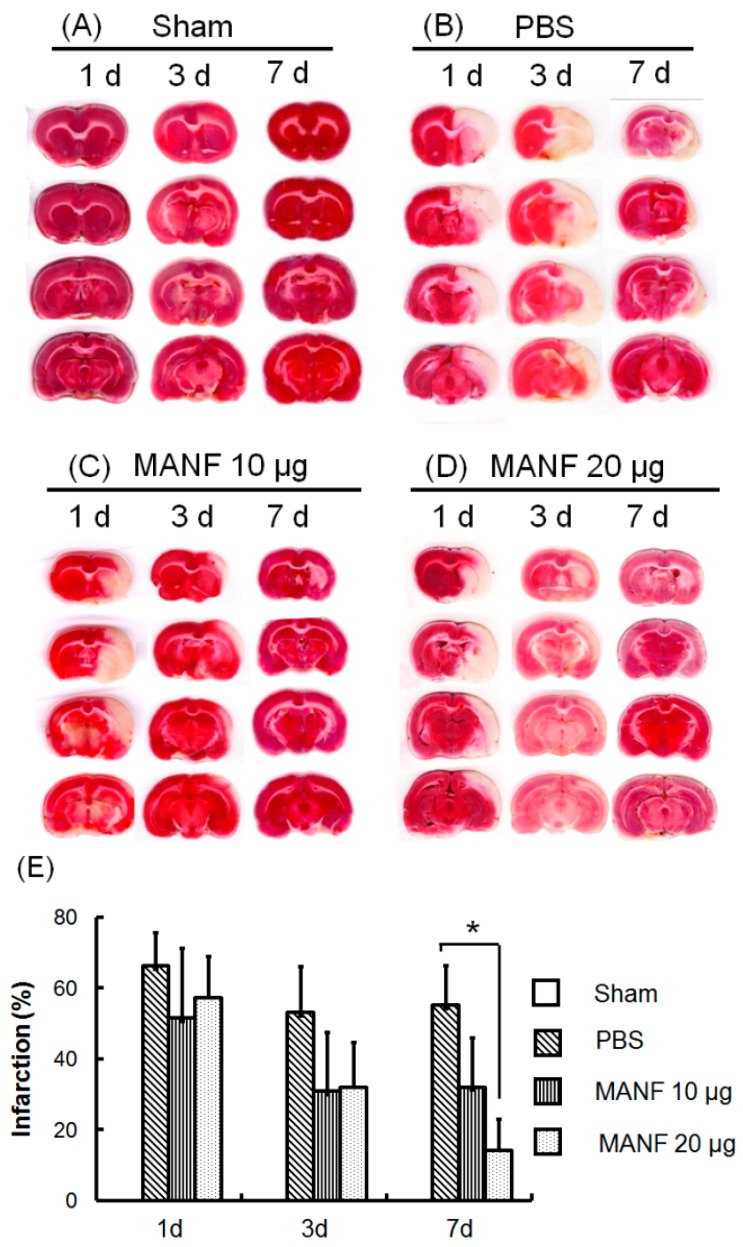
TTC staining images of the brain sections of a representative rat in the sham group (**A**), the PBS group (**B**), the 10-μg MANF group (**C**), and the 20-μg MANF group (**D**); (**E**) the corresponding infarct percentage. The quantitative values are expressed as mean ± SD. * *p* < 0.05, compared with the PBS group. d: day.

TTC staining was used to confirm the therapeutic effect of MANF on focal cerebral ischemia, which was dynamically evaluated by MRI. The brain sections of a representative rat in each group are shown in Figure 5. The infarct tissue appeared to be a white area, and the non-infarct tissue was pink. The tissues in both hemispheres in the sham group are the same. In the PBS group, the tissue was necrotic, which made the ipsilateral hemisphere shrink, while in the MANF group the volume of necrotic tissue was significantly reduced. The quantitative analysis on the percentage of infarct volume showed that the brain injury was relieved in the MANF group compared with the PBS group. The difference was significant on Day 7 after treatment.

### 2.5. MRI Manifestation Was Confirmed by Histologic Analyses in Evaluating the Therapeutic Effect of MANF on Focal Cerebral Ischemia

**Figure 6:**
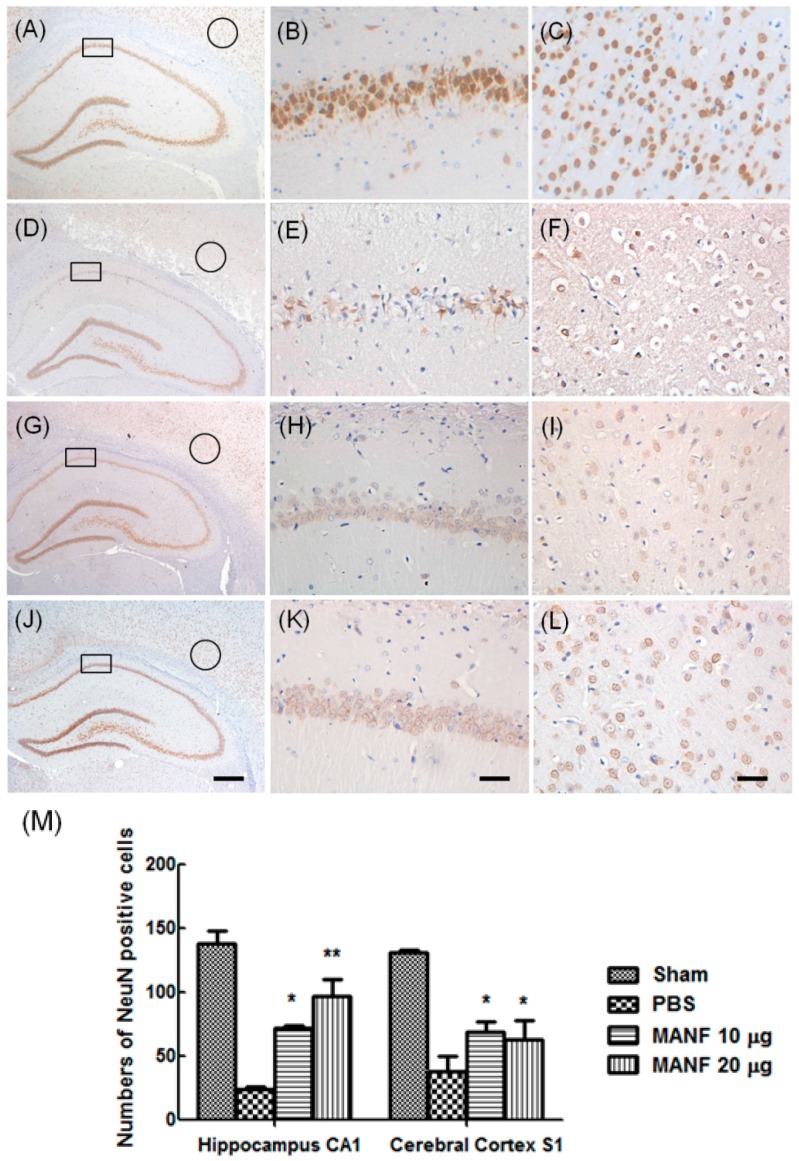
Assessment of ischemic lesions by histology. NeuN-positive DAB staining images collected from brain sections of a representative rat in the sham group (**A**–**C**), the PBS group (**D**–**F**), the 10-μg MANF group (**G**–**I**), and the 20-μg MANF group (**J**–**L**). The rectangle areas in **A**, **D**, **G**, and **J** are shown in higher magnification in **B**, **E**, **H**, and **K**, respectively. The circular areas in **A**, **D**, **G**, and **J** are shown in higher magnification in **C**, **F**, **I**, and **L**, respectively. The scale bars in **A**, **D**, **G**, and **J** are 400 µm; while in other photographs they are 40 µm. The number of NeuN-positive cells were counted and quantitatively analyzed, as shown in Figure **M**. Data are presented as means ± SD. * *p* < 0.05, ** *p* < 0.01, compared with PBS controls.

To confirm the therapeutic effect of MANF on neuron survival after MCAO, the expression of neuron marker NeuN was detected by using of immunohistochemistry staining (Figure 6). It had been demonstrated that loss of NeuN immunoreactivity occurred with CNS injury [23]. The brain was taken 3 days after MANF protein administration. In the PBS group, the nucleus size of NeuN-positive neurons decreased in the cerebral cortex and hippocampus, compared with the sham control (Figure 6D–F). There were also a large number of neurons lost in the PBS group (Figure 6M). In the MANF-treated groups, there was no significant difference in the number of neurons compared to the contralateral. The number of NeuN-positive neurons in the MANF groups was larger than that in the PBS group. These results are consistent with the manifestation in MRI, which also suggest that MRI is an effective method to evaluate the therapeutic effect of MANF on focal cerebral ischemia.

## 3. Discussion

Several studies about the protective effects of MANF on neuron lesion induced by ischemia have been recently reported. However, among this research, some focused on the effect of endogenous MANF, and some focused on the neuroprotection of MANF with pretreatment. Additionally, the effect of MANF was evaluated at a single time point, which may not completely reflect the profile of MANF’s neuroprotection. In the present study, we dynamically observed the effects of MANF on ischemia/reperfusion-induced injury at the different time points by MRI, especially in reperfusion phase.

In the previous studies, MANF was administrated before reperfusion injury. This strategy has less clinical significance, because deleterious biochemical processes can be triggered during reperfusion after ischemia. The injury after reperfusion is usually obvious due to secondary hemo-dynamic disturbances, the enhancement of inflammatory process, free radical formation, vasogenic edema, and the breakdown of the blood brain barrier (BBB). Therefore, choosing the proper time window for therapy is very important.

In the clinic, T1, T2, and diffusion-weighted imaging (DWI) are common methods to diagnose ischemic stroke. DWI is very sensitive for acute cerebral ischemia. However, it tends to overestimate the ischemic lesion in subacute ischemia because of the changes in the diffusion status of tissue [18]. Although T2 is considered less useful in the early stage following stroke since it apparently increases only later in the disease process, it reflects the changes in tissue water content associated with vasogenic edema and is better at predicting the change of infarct over a longer period of time. It is currently not well understood when T2-weighted imaging changes occur within the early stage. Such information will be important for diagnosis and the following therapy. In these cases, T2-weighted imaging is performed. At the early stage of ischemia/reperfusion injury, the increase in T2 signal is indicative of edema and breakdown of the BBB. In the present study, the obvious cerebral edema was found by MRI at 3 h after reperfusion in all experimental rats. Based on MRI investigation, the time point chosen for MANF protein administration is at 3 h after reperfusion.

Many studies have shown that the neurological function after stroke was recovered spontaneously without treatment. In this work, we also found that ischemia lesion size decreased with varying degrees in all experimental groups from 2 to 7 days by MRI. MANF protein decreased the mortality of MCAO rats within the early period of ischemic injury, and constantly lowered the ischemia lesion size shown by MRI images. In the ischemia/reperfusion rats, the signal changes in T2 images are mainly attributed to the vasogenic edema. This kind of edema may be caused by the injury of the BBB, which let macromolecules and water enter into the ischemic tissue. During the long-term observation, the overall lesion volume in the MANF-group rats was signiﬁcantly smaller than that in the PBS-group rats. The infarct volume and percentage measured by TTC staining were slightly larger than that measured by T2-weighted images, which may be explained by the fact that TTC staining may not represent the irreversible cell death and thus may overestimate infarct size [24].

With respect to the body weight, all three experimental groups showed an initial decrease on the first day after MANF treatment. This change may be attributed to the surgery injury resulted from MCAO and intracerebroventricular injection. After that, the body weight of rats administrated with MANF protein exhibited an increase compared with rats treated only with PBS, which indicates that the MANF protein makes crucial contributions for the therapy of cerebral ischemia/reperfusion injury.

From the view of neurological function test, although the Bederson’s score of the rats in the MANF-treated group was zero, suggesting that MANF protein has a therapeutic effect on ischemia/reperfusion injury, the lesion area was still observed in both T2-weighted images and TTC-stained sections. This kind of difference is deemed acceptable, since the neurological function tests are not an accurate method.

As for the positive control, we selected edaravone, but not t-PA. Although t-PA is the only thrombolytic agent approved by the Food and Drug Administration (FDA), its function is to dredge the occlusive vasculatures and increase cerebral blood flow in ischemic regions [3], which is not suitable for the MCAO model because the vasculature had been dredged by reperfusion. Edaravone (3-methyl-1-phenyl-2-pyrazolin-5-one) is a free radical scavenger and has neuroprotective effects on acute cerebral infarction, which has been approved by Japanese health authorities since 2001 [25]. The effect of edaravone was investigated by MRI, which is shown in Figure S1. Although MANF protein has a weaker effect on ischemia/reperfusion injury than edaravone, MANF protein still plays an important role in reliving the injury and reducing the infarct area. MANF may exert its neuroprotection along with other clinical agents to obtain a synergy effect.

It has been well-known that caspase-3 plays a critical role in the common pathway of apoptosis. Our previous study [10] demonstrated that cerebral ischemia/reperfusion injury caused by MCAO triggered the cleavage of caspase-3, and the level of the cleaved caspase-3 was suppressed by MANF treatment, which suggests that the neuroprotective effects of MANF in the ischemic cerebral cortex may be intermediated by inhibiting cell apoptosis. Our study also showed MANF inhibited IRE1-XBP1 pathway activation, but did not affect XBP1 transcription [14], which suggests that MANF protects neurons from loss via regulating UPR-related genes. It has also been well known that the inflammatory process is involved in ischemia-induced neuronal injury. We found that MANF was upregulated in the activated glial cells [26]. Joana Neves et al. recently identified immune cells as a target for MANF [27]. We also showed that MANF can further negatively regulate nuclear factor κB (NF-κB) signaling in mammalian cells [28]. These findings may contribute to the mechanisms underlying ischemia-induced neural injury.

## 4. Materials and Methods

### 4.1. Materials

Recombinant human MANF protein was expressed and purified according to a method described previously [13]. Briefly, recombinant MANF protein was expressed in *E. coli* BL21 using 1 mM IPTG induction and purified using a Ni^2+^-affinity column. The purity was evidenced by SDS-PAGE (Figure S2), and the concentration was measured by BCA kit (Solarbio, Beijing, China). All the chemicals were purchased from Sigma-Aldrich (St. Louis, MO, USA.).

### 4.2. Animals

Male Sprague Dawley (SD) rats (6–8 weeks old, weighing 200–220 g) were obtained from the Anhui Experimental Animal Center and housed in SPF conditions. The animal license number is SCXK (Wan) 2011-002. The rats were housed under a controlled temperature (22 ± 1 °C) with a 12-h light/dark cycle and were allowed free access to food and water. All the experimental procedures for animal surgery were approved by the Ethics Committee of Anhui Medical University for Animal Experimentation and performed in accordance with the Guideline of the Declaration of Helsinki.

### 4.3. Induction of Focal Cerebral Ischemia/Reperfusion Injury

Focal cerebral ischemia was induced by MCAO as described previously [14]. The rats were anesthetized with 10% chloral hydrate by intraperitoneal injection. Briefly, a 2-0 nylon monofilament suture, blunted at the tip and coated with nail oil, was inserted through the right common carotid artery into the internal carotid artery and advanced approximately 18 ± 0.5 mm distal to the carotid bifurcation to occlude the origin of the middle cerebral artery at the junction of the circle of Willis. Ninety minutes after occlusion, the suture was withdrawn to allow reperfusion. Sham-operated animals were subjected to the same procedure, except the artery was not occluded. After surgery, the rats were kept in a warm box heated by lamps until they woke up and then returned to their home cages.

### 4.4. Drug Administration

The animals were randomly divided into 4 groups with a random number table after surgery: sham group (*n* = 6), PBS group (control group, *n* = 14), and MANF groups with a low dose (10 μg, *n* = 9) and a high dose (20 μg, *n* = 9). MANF protein was injected intracerebroventricularlly at 3 h post-reperfusion. MANF solution (20 μL, 0.5 or 1 μg/μL) or an equal volume of PBS solution was injected into the right lateral ventricle with a coordinate of 1.5 mm lateral to the midline, 1.0 mm posterior to the bregma, and 4.5 mm deep at a rate of 1 μL/min. The needle was retained in place for 5 min after injection.

### 4.5. Neurological Function Test

The neurological function was tested by using Bederson’s score [23]: 0: rats extend both forelimbs straight without observable deficit; 1: rats keep the one forelimb to the breast and extend the other forelimb straight; 2: rats show the decreased resistance to lateral push in addition to behavior in score 1 without circling; 3: rats twist the upper half of their body in addition to behavior in score 2.

### 4.6. Magnetic Resonance Imaging

Before imaging, the rats were anesthetized as described earlier and placed into the bore of the MRI magnet in a prone position. MRI was performed on a 3.0-T GE Sigma HDX MRI scanner (GE, Milwaukee, WI, USA) by using a 4-channel animal coil. Each MRI session consisted of 10 transverse T2-weighted slices. T2-weighted images were obtained from a 2.0-mm-thick coronal section with a 60 mm × 48 mm field of view, TR = 2440 ms, TE = 106.6 ms, and a matrix of 320 × 320. The lesion volumes were determined by an investigator experienced in experimental stroke MRI with the image processing software (Chris Rorden’s MRIcro 2.0, www.mricro.com). The edge of the lesions was drawn manually on each of the slices, and the areas were then summed and multiplied by the slice thickness to calculate the infarct volume.

### 4.7. 2,3,5-Triphenyltetrazolium Chloride (TTC) Staining

After the rats were decapitated, the brains were removed and sliced into 2.0-mm-thick sections. The brain slices were incubated in a 2% TTC solution (Sigma, St. Louis, MO, USA) for 30 min at 37 °C and then transferred into a 4% paraformaldehyde solution for fixation. TTC-stained sections were captured using a digital scanner (HP Scanjet 200, Hewlett-Packard Development Company, Palo Alto, CA, USA) and the area of infarction in each slice was measured with Image J software (Wayne Rasband, National Institutes of Health, Bethesda, MA, USA). In order to minimize the error caused by brain edema, the total infarct volume and infarct percentage were manually calculated according to the following formulas [29,30]:

Infarct volume=contralateral hemisphere region−non-infarcted region in the ipsilateral hemisphereInfarct percentage=infarct v/v of the contralateral hemisphere×100%

### 4.8. Immunohistochemistry

Brain tissues were fixed with 4% paraformaldehyde. Serial coronal sections (3 μm) were prepared. The sections were incubated with primary antibodies overnight at 4 °C after blocking by 5% goat serum. The sections were then incubated with secondary antibodies at 37 °C for 1 h. 3′-3′-diaminobenzidine tetrahydrochloride (DAB) solution was applied to the sections and allowed to react for 15 s. The sections were stained with hematoxylin, dehydrated in ethanol, and mounted in neutral gum. The number of NeuN-positive cells in cerebral cortex S1 and hippocampus CA1 was counted in five randomly selected fields in one section, and the average was calculated. Four sections were used in each group. The average from four sections was used to calculate the mean and SD of each group for further statistical analysis.

### 4.9. Statistical Analysis

All data were expressed as mean ± standard deviation (SD). Statistical comparisons were analyzed by using a one-way ANOVA with Dunnett’s test. *p* < 0.05 was considered statistically significant.

## 5. Conclusions

The present study uniquely reported a dynamic MRI study that monitors and evaluates the effect of MANF protein on ischemia/reperfusion injury in rats with MCAO. Our data demonstrate that intracerebroventricular injection of MANF protein at 3 h after reperfusion can reduce the infarct volume, increase the survival rate after ischemia/reperfusion injury, and speed up the locomotor functional recovery. Although MANF protein has not cured the ischemia/reperfusion injury completely, it relives the injury and reduces the lesion area, which may become a potential choice for the combined therapy of ischemia brain injury in the future.

## Supplementary Materials

Supplementary materials can be found at www.mdpi.com/1422-0067/17/9/1476/s1.
