# Supplementary material for: MRI Dynamically Evaluates the Therapeutic Effect of Recombinant Human MANF on Ischemia/Reperfusion Injury in Rats
Source: Int J Mol Sci. 2016 Sep 5;17(9):1476. doi: 10.3390/ijms17091476 (PMC5037754; doi:10.3390/ijms17091476)
Supplement: Supplementary file 1 [file ijms-17-01476-s001.pdf]

Supplementary Materials: MRI Dynamically Evaluates the Therapeutic Effect of Recombinant Human MANF on Ischemia/Reperfusion Injury in Rats

Xian-Yun Wang, Meng-Meng Song, Si-Xing Bi, Yu-Jun Shen, Yu-Xian Shen and Yong-Qiang Yu

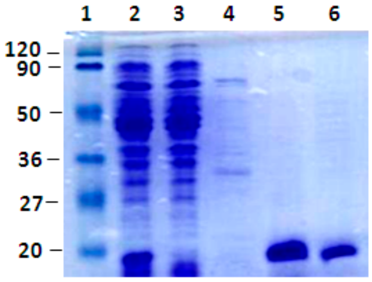

Lane 1: marker
Lane 2: whole lysate
Lane 3: nonbound components of the lysate
Lane 4: collected washing buffer
Lane 5: collected elution buffer containing 200 mM imidazole
Lane 6: collected elution buffer containing 300 mM imidazole

Figure S1. SDS-PAGE gel for detection of purified His-mesencephalic astrocyte-derived neurotrophic factor (MANF).

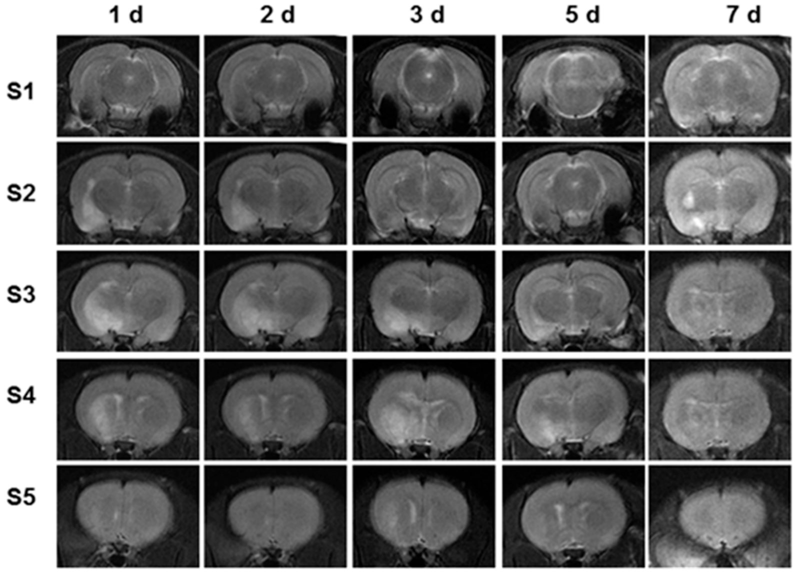

Figure S2. T2-weighted images of 5 brain slices (S1–S5) from a representative rat in edaravone group 1, 2, 3, 5, and 7 days after treatment. Rats were treated with tail vein infusion of edaravone at 3 mg/kg body weight twice: immediately and 30 min after reperfusion of middle cerebral artery occlusion (MCAO). This treatment shecule and dosage were based on the the pharmacokinetic profile of edaravone supplied by the manufacturer. d: day.
